# How do humans inspect BPMN models: an exploratory study

**DOI:** 10.1007/s10270-016-0563-8

**Published:** 2016-10-07

**Authors:** Cornelia Haisjackl, Pnina Soffer, Shao Yi Lim, Barbara Weber

**Affiliations:** 1 University of Innsbruck, Innsbruck, Austria; 2 University of Haifa, Haifa, Israel; 3 Technical University of Denmark, Kongens Lyngby, Denmark

**Keywords:** Process model quality, Process model maintainability, Empirical research, Human-centered support

## Abstract

Even though considerable progress regarding the technical perspective on modeling and supporting business processes has been achieved, it appears that the human perspective is still often left aside. In particular, we do not have an in-depth understanding of how process models are inspected by humans, what strategies are taken, what challenges arise, and what cognitive processes are involved. This paper contributes toward such an understanding and reports an exploratory study investigating how humans identify and classify quality issues in BPMN process models. Providing preliminary answers to initial research questions, we also indicate other research questions that can be investigated using this approach. Our qualitative analysis shows that humans adapt different strategies on how to identify quality issues. In addition, we observed several challenges appearing when humans inspect process models. Finally, we present different manners in which classification of quality issues was addressed.

## Introduction

Much conceptual, analytical and empirical research has been conducted during the last decades to advance our understanding of conceptual modeling. Specifically, process models have gained significant importance in recent years due to their critical role for the management of business processes [4]. Business process models help to obtain a common understanding of a company’s business processes [45], serve as drivers for the implementation and enactment of business processes and enable the discovery of improvement opportunities [48]. Even though considerable progress regarding process modeling languages and methods has been achieved, the question how *humans* can be efficiently supported in creating, understanding and maintaining business process models is still a lingering problem. One consequence is that process models still display a wide range of quality problems impeding their comprehension and maintainability [58]. Similarly, literature reports on error rates between 10 and 20 % in industrial process model collections [29]. Moreover, process model smells like non-intention-revealing or inconsistent labeling [30] are typical quality issues, which can be observed in existing process model collections. In addition, layout conventions are often missing [46], resulting in models that lack a consistent graphical appearance, thereby introducing an additional burden for humans when building an understanding of process models.

While some quality issues, mostly syntactic, can be detected automatically by verification algorithms (e.g., [61]), many others cannot. Hence, human inspection of process models is still essential [53]. This manual inspection is currently not supported. Furthermore, we do not have an in-depth understanding of how it is conducted, what strategies are taken, what challenges arise, and what cognitive processes are involved. In addition, whether and how awareness of a classification of quality issues supports this process is an open question.

Taking a first step toward such an understanding, this paper reports a study which explores the model inspection process (extending results from [18]). When exploring a question which has not been addressed so far, a main issue is to identify an appropriate research approach and show that it can be applied to the current question. In the reported study, we investigate the strategies taken by humans when inspecting a process model, the kinds of challenges that appear during this process and different manners in which classification is addressed.

In order to tackle these research objectives, an exploratory study utilizing *think-aloud* is conducted, asking humans to find different types of quality issues, i.e., syntactic, semantic and pragmatic, in process models of varying sizes and to classify them. By analyzing the think-aloud protocols, we were able to identify different strategies how subjects inspect process models. Further, we observed that for each quality dimension of the SEQUAL [25] framework quality problems were spotted by a large number of subjects, but also quality issues that gained less attention. This paper extends the work presented in [18] by including results of a substantial additional analysis of the data collected in the study, yielding insights that were not included in [18]. For instance, we identified several challenges which explain why subjects on the one hand were not able to spot quality problems and, on the other hand, marked issues that were neither errors nor process model smells. In particular, we could trace challenges back to lack of BPMN knowledge, lack of domain knowledge, unclear inspection criteria, problems with the context of quality problems and the trouble to simply overlook quality issues. Additionally, we observed different manners relating to the classification of quality issues. We noticed that even though most quality problems were classified correctly, some subjects had problems to differentiate between semantic and pragmatic quality issues. Our findings contribute toward a better understanding of how humans inspect process models, guiding model inspection support for humans as well as pointing out typical challenges to teachers and educators of future system analysts.

The remainder of the paper is structured as follows: Sect. 2 gives background information. Section 3 describes the setup of the study, whereas Sect. 4 deals with its execution. Section 5 presents the findings of the study, and Sect. 6 a corresponding discussion. Related work is presented in Sect. 7. Finally, Sect. 8 concludes the paper.

## Background

This section discusses different types of quality issues along the dimension of the SEQUAL [25] framework, i.e., syntactic, semantic, and pragmatic quality. This classification of quality issues was used for the exploratory study described in Sect. 3.

### Syntactic quality

Syntactic quality refers to the correspondence between the model and the language the model is described in. Different views exist in the literature on how to categorize quality issues that are resulting from a violation of the soundness property like deadlocks or the lack of synchronization (e.g., [20, 26, 41, 53]). Classification used for this study subscribes to the view of [20, 41] that soundness can be attributed to the syntactic layer of the SEQUAL framework. By giving specific examples as part of the task description, we aimed to ensure that subjects are adhering to the same view. Note that most syntactic errors can be detected automatically. Existing works have analyzed typical syntactical errors at IBM [24], in the SAP reference model [28] and other large industrial model collections [47].

### Semantic quality

Semantic quality refers to both the validity (i.e., statements in the model are correct and related to the problem) and the completeness (i.e., the model contains all relevant and correct statements to solve this problem) of the model. Invalid behavior and superfluous activities are typical examples violating the validity of a process model. Typical errors regarding the completeness of a model include missing activities. Semantic quality issues have been addressed only to a limited extent so far [54] and can only be identified by human.

### Pragmatic quality

Finally, pragmatic quality can be described as the correspondence between the model and people’s interpretation of it, which is typically measured as model comprehension [25]. Significant research has been conducted in recent years on factors that impact process model comprehension, such as the influence of model complexity [44], modularity [63, 64], grammatical styles of labeling activities [30] and secondary notation [50]. Respective insights led to the development of empirically grounded guidelines for the modeling of business processes [32], describing process model smells [32, 58]. Examples of process model smells are non-intention-revealing names of activities [58], crossing edges [40] or reverse sequence flow direction [17]. While some of these issues can be handled automatically [58], a comprehensive quality inspection by human is still essential.

## Designing the exploratory study

Despite the importance of human inspection of process models, there has been no considerable research on how subjects identify quality issues. Hence, since no theories exist we can base our investigation on, we address the topic in an exploratory manner using a qualitative research approach [2]. In particular, we use the think-aloud method, i.e., we ask participating subjects to voice their thoughts, allowing for a detailed analysis of their reasoning process [14]. Then, we turn to grounded theory [9], where theory emerges when analyzing data, identifying recurring aspects and grouping them to categories. These categories are validated and refined throughout the analysis process. First of all, we describe setup and planning of the exploratory study.

### Research questions

The goal of this study is to gain in-depth understanding how humans identify quality issues in process models to guide model inspection support for humans. In addition, typical challenges of this process should be pointed out to teachers and educators of future system analysts. The research questions as well as most of our findings are generic to imperative modeling languages. This is also supported by [43], which presents a study showing that EPC users understand BPMN diagrams equally well even though they were never exposed to this modeling language before. BPMN was selected as a representative process modeling language due to its prevalence and wide acceptance as a de facto standard [42]. In particular, we are interested in common strategies that humans apply for identifying quality issues. Research question $RQ_{1}$ can be stated as follows:

**Research Question** ${{\varvec{RQ}}}_{\mathbf{1}}$ *What are common strategies that humans take for inspecting and identifying quality issues in BPMN process models?*

Further, process model inspection is not easy and might raise challenges. While focusing on BPMN, we expect some of these challenges to be generic while some would be BPMN specific. In addition, the challenges might vary for different types of quality problems, i.e., syntactic, semantic, and pragmatic quality issues. Therefore, in research question $RQ_{2}$ we investigate challenges humans face when inspecting a process model:

**Research Question** ${{\varvec{RQ}}}_{\mathbf{2}}$ *What are the challenges in identifying quality problems in BPMN process models?*

The investigated task relies on human cognitive processes. We sought for effective yet applicable ways of supporting these processes. Therefore, we turn to the use of classification, which has been found effective for conceptualization in general, and conceptualizing process behavior in particular [55]. Classification supports such tasks by providing a structured and ready-to-use model by which observed phenomena can be tagged and interpreted, prompting additional conclusions that can be drawn [37]. Hence, we investigate how classifying spotted quality problems according to the dimensions of the SEQUAL [25] framework was done and whether there is evidence that classification indeed helped:

**Research Question** ${{\varvec{RQ}}}_{\mathbf{3}}$ *Can classifying issues with the quality dimensions of the SEQUAL framework help humans when inspecting a BPMN process model?*

### Subjects

In order to ensure that obtained results are not influenced by unfamiliarity with BPMN process modeling, subjects need to be sufficiently trained. Even though we do not require experts, subjects should have at least a moderate understanding of imperative processes’ principles. For information on the actual subjects, see Sect. 4.

### Objects

Since we were interested in how subjects identify quality issues, we created two BPMN models ($P_1$ and $P_2$) from informal process descriptions and introduced several quality issues. The process models cover all essential control flow patterns as well as message and timer events [33]. Table 1 summarizes the characteristics of the process models. They vary regarding the amount of these modeling elements, e.g., the number of activities (between 13 and 45) and number of message flows (between 4 and 8) which are realistic sizes for business process models [15]. The process models are based on two different domains describing an order-to-cash process (i.e., a company selling self-mixed muesli) [13] and a new product development process.

**Table 1:** Characteristics of $P_1$ and $P_2$

	$P_1$	$P_2$
Number of pools	2	2
Number of activities	13	45
Number of gateways	10	16
Number of message events	8	1
Number of timer events	3	1
Number of edges	41	76
Number of messages flows	8	4

The process models do not only cover all essential modeling elements, but also relevant quality issues of all three dimensions, i.e., syntactic, semantic, and pragmatic (cf. Sect. 2). Table 2 summarizes these introduced quality problems. $P_1$ and $P_2$ comprise between 5 and 9 syntactic, between 3 and 4 semantic, and between 6 and 12 pragmatic quality issues. In particular, syntactic issues cover, for instance, usage of wrong modeling elements and deadlocks. Regarding semantic quality, validity is offended by invalid behavior (i.e., it can happen that the customer has to pay more than once for a product), superfluous activities (e.g., activity “Call Claudia”), and switched lane labels. Since the subjects are not domain experts, incompleteness could not be introduced to the process models. Relating to pragmatic issues, for example, label issues (e.g., non-intention-revealing labels), line crossings, and reverse sequence flow direction were introduced.^1^

**Table 2:** Number of quality issues in $P_1$ and $P_2$

	$P_1$	$P_2$
Syntactic issues	5	9
Wrong modeling element usage	4	4
Missing transition conditions	0	1
Deadlock	1	2
Livelock	0	1
Lack of synchronization	0	1
Semantic issues	3	4
Superfluous activity	2	3
Invalid behavior	1	0
Switched lane labels	0	1
Pragmatic issues	6	12
Label issues	2	3
Line crossings	1	2
Message flow descriptions	1	0
Compact layout	1	0
Erratic sequence flow direction	1	0
Reverse sequence flow direction	0	3
Implicit gateways	0	2
Crooked alignment	0	2
All issues	14	25

**Fig. 1:**
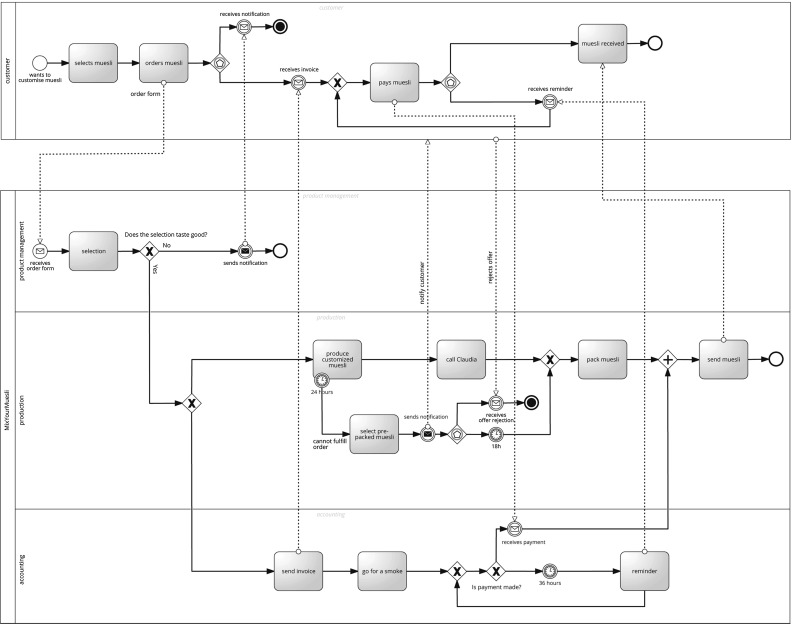
Process model $$P_1$$

For example, Fig. 1 shows process model $P_1$.^2^ The model consists of two pools. The first one represents the customer, and the second one comprises the muesli mixing company.

### Design

**Fig. 2:**
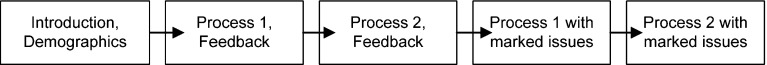
Design of the exploratory study

Figure 2 shows the overall design of the study: First, subjects obtain introductory assignments and demographic data are collected. Afterward, subjects are confronted with the actual tasks. Each subject works on two process models. For each model, the subjects are asked to find as many quality issues as they can. As we are also interested if classification is helpful, the subjects should classify each spotted quality issue according to the dimensions of the SEQUAL [25] framework. The different dimensions are listed at the task description in the introductory assignments. In addition, small examples are given, e.g., deadlocks and lack of synchronization for syntactic quality. However, as we are interested in the way subjects learnt, understood and expressed this classification, giving too detailed descriptions would influence the subjects. After each model, we ask for assessment of mental effort as well as for feedback on domain knowledge, understandability of the model, difficulties with think-aloud, and difficulties understanding the model’s language (English). Finally, subjects are shown the same process models with marked quality issues and are asked to comment on the quality issues they were not able to find.

### Instrumentation

For each model, subjects received separate paper sheets showing the process models, allowing them to use a pen for highlighting quality issues or write down the issue’s classification. No written answers were required, only free talking. Audio and video recording are used as it has proven being useful for resolving unclear situations in think-aloud protocols [62].

## Performing the exploratory study

### Execution

The study was conducted in October and December 2014 at the University of Innsbruck, i.e., a total of twelve subjects participated. This number is considered appropriate for think-aloud studies due to the richness of the collected data [10, 35]. Each session was organized as follows: First, the subject was welcomed and instructed to speak thoughts out loudly. One supervisor handed sheets containing the study’s material out and collected them as soon as the subject finished the task. Meanwhile, the subject’s actions were audio- and video-recorded to gather any uttered thoughts.

### Data validation

In each session, only a single subject participated, allowing us to ensure that the study setup was obeyed. In addition, we screened whether subjects fitted the targeted profile, i.e., were familiar with process modeling and BPMN. We asked questions regarding familiarity with process modeling, BPMN, and domain knowledge; note that the latter may significantly influence performance [23]. For this, we utilize a 7-point Likert scale, ranging from “Strongly agree” (7) over “Neutral” (4) to “Strongly disagree” (1). Results are summarized in Table 3. Finally, we assessed the subjects’ professional background: Six subjects were students, four subjects had an academic background (i.e., were either PhD students or postdocs), and two subjects indicated a professional background. We conclude that all subjects had an adequate background in process modeling (the least experienced subject had 2 years of modeling experience) and were moderately familiar with BPMN.

**Table 3:** Demographics (5–9 based on 7-point Likert scale)

	Minimum	Maximum	Median
1) Years of modeling experience	2	6	4
2) Models read last year	2	250	5
3) Models created last year	0	50	2.75
4) Average number of activities	0	30	11
5) Familiarity BPMN	2	6	5
6) Confidence understanding BPMN	3	6	6
7) Confidence creating BPMN	3	6	5.5
8) Familiarity mixing muesli company	5	7	6
9) Familiarity new product development company	2	7	5

### Data analysis

Our data analysis comprised the following stages. As a starting point, we transcribed the subjects’ verbal utterances. Afterward, we applied grounded theory methods to the transcripts in order to answer our research questions.

#### $RQ_{1}$

To investigate what kind of strategies the subjects applied to identify quality issues, we inspected the transcripts, marking aspects that pointed to the usage of a strategy. An example for such an aspect would be a subject indicating to get an overview of the process model before inspecting it. In a second iteration, we revisited the marked areas and searched for new aspects. This process of open coding analysis was repeated until no new aspects could be found, so saturation has been reached. Afterward, we performed axial coding, i.e., we repeatedly grouped aspects to form high-level categories. We counted the number of identified markings per category, i.e., the number of subjects belonging to one of the three identified strategies (cf. Sect. 5.1).

**Fig. 3:**
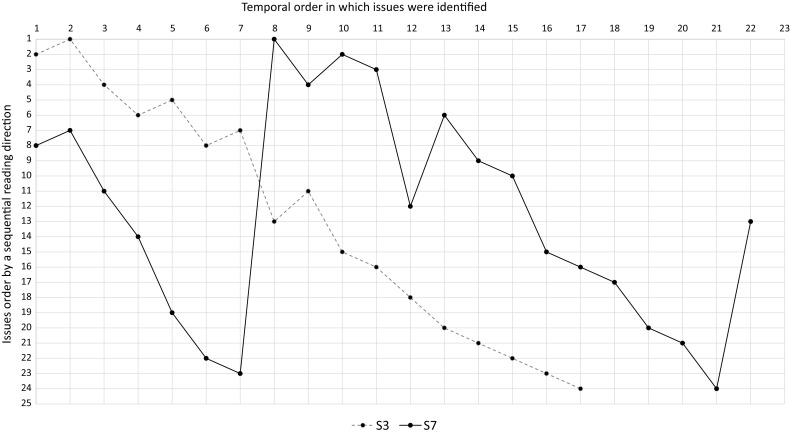
Temporal-order issues were identified in $P_2$ with $T_1$

**Fig. 4:**
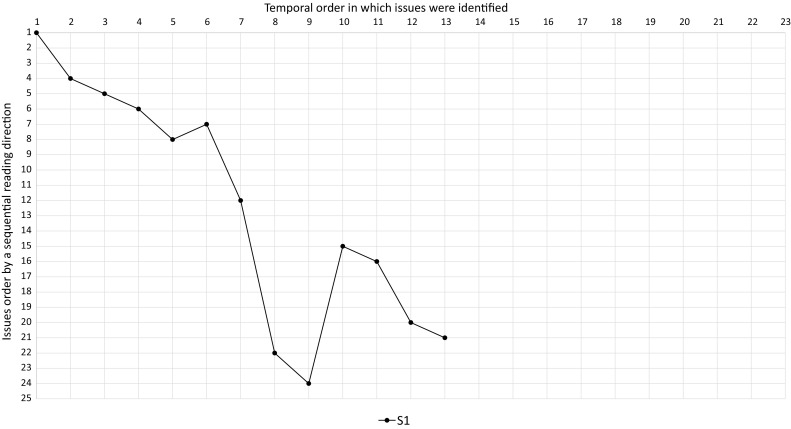
Temporal-order issues were identified in $P_2$ with $T_2$

**Fig. 5:**
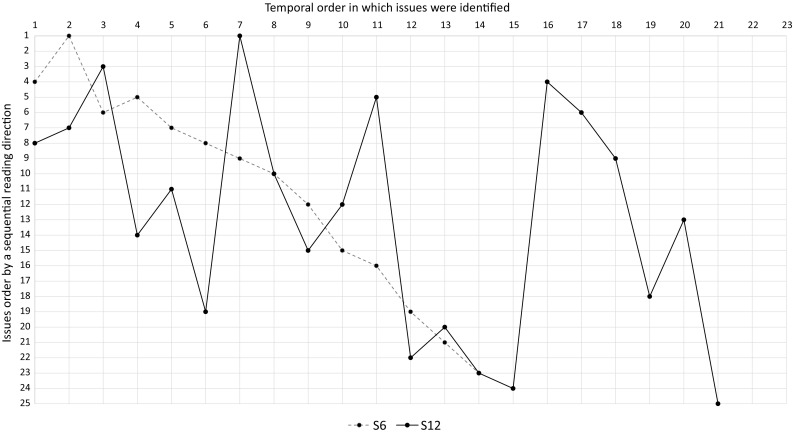
Temporal-order issues were identified in $P_2$ with $T_3$

To obtain more detailed insights into the strategies subjects applied, we also investigated the reading direction or temporal order of issue identification that humans adopt when inspecting process models. Therefore, we ordered the quality issues of each model according to a sequential way of reading the specific model. Since several alternative ways of sequentially reading each model exist, we chose one specific sequential ordering that conforms to the reading direction the majority of the subjects chose. This allowed us to visualize the way in which subjects inspected the models in diagrams (cf. Figs. 3, 4, 5). The horizontal axis displays the temporal order in which issues were identified, while the vertical axis shows all issues sorted according to our sequential reading order. If a subject would have identified issues in exactly this sequential reading order, the diagram would show a linear line between point 1,1, and point 25,25. Afterward, we extracted the temporal issue identification order for each subject from the transcripts to fill the diagrams with data. In addition, we counted how often subjects iterated over the models and how many issues (i.e., true positives) were found during these iterations (cf. Table 4).

#### $RQ_{2}$

To examine challenges that appear when identifying quality problems in process models, we observed the transcripts’ part of subjects inspecting the models as well as the transcripts’ part of subjects commenting the models with highlighted quality issues. We marked all parts of the transcripts relating to quality issues and thereby differentiated between true positives (correctly identified quality problems), false negatives (quality issues present in the models that were not mentioned by subjects), and false positives (marked issues that are neither errors nor process model smells, which further will be referred to as non-issues). Again, the process of open coding was repeated until saturation has been reached. Like before, we performed axial coding, i.e., we repeatedly grouped aspects to form high-level categories describing challenges. Finally, we counted the number of identified markings per category (cf. Sect. 5.2).

#### $RQ_{3}$

As mentioned in Sect. 3, we asked subjects to classify marked quality problems along the dimensions of the SEQUAL framework, i.e., syntactic, semantic, and pragmatic. To answer the question if the classification is helpful, we applied grounded theory methods to these comments, i.e., we focused on aspects that indicate how classification was done. Again, we repeatedly grouped these aspects to form high-level categories. In addition, we inspected if quality problems were classified correctly (cf. Sect. 5.3).

## Findings

In the previous section, we have discussed the design and execution of the study. In the following, we use the gathered data to investigate research questions $RQ_{1}$ to $RQ_{3}$.

### $RQ_{1}$: What are common strategies that humans take for inspecting and identifying quality issues in BPMN process models?

When analyzing the transcripts, we observed that subjects consistently adopted similar strategies while identifying quality issues in BPMN process models.^3^ We could identify three different strategies:

**Table 4:** Number of correctly identified quality issues (i.e., true positives), spotted by subjects applying $T_1$

	S2	S3	S4	S5	S7	S8	S9	S10	S11
$P_1$ overview	–	0	–	0	0	0	0	0	0
$P_1$ 1st iteration	–	2	–	8	6	7	5	6	3
$P_1$ 2nd iteration	–	3	–	1	0	0	0	–	–
$P_1$ 3rd iteration	–	0	–	–	–	–	–	–	–
$P_2$ overview	0	0	1	1	7	0	0	0	1
$P_2$ 1st iteration	14	18	14	14	15	16	13	18	15
$P_2$ 2nd iteration	–	–	–	0	1	–	–	–	–

#### Strategy $T_1$

The majority of subjects identified quality issues in the model by first getting an overview of the model and then checking the whole model for quality issues. For $P_1$ 7 out of 12 subjects (58.33 %) and for $P_2$ additional 2 subjects (9 out of 12 subjects, 75.00 %) used strategy $T_1$. In detail, there are three ways how subjects overviewed the model. First, they started by reading the pool and lane descriptions. Second, they read through the whole model before they checked it for any quality issue. Third, they looked at the structure of the process model. Relating to $P_1$ depicted in Fig. 1, 4 subjects listed the pools (e.g., “First I’m looking at the different kinds of lanes and pools...So apparently it’s the company ‘Mix Your Muesli’ and the customer.”), 2 subjects read silently the whole model, and one subject glanced over the structure of the model (“Ok, so...first of all I look at the structure.”). Afterward, they started reading the model while looking for quality issues: “Let’s start at the start point. The customer wants to customize muesli...Ok, this is the start event.”

The order in which errors were identified is closely related to the respective strategy. Therefore, subjects that adopted $T_1$ started by getting an overview of a model. Then they inspected the model in up to 3 iterations, i.e., starting with the start event, they read the model following the sequence or message flows. Table 4 shows the number of true positives identified per iteration by subjects applying $T_1$. For $P_1$, no issues were found while getting an overview of the model. Nearly all issues were identified during the first iteration, i.e., only 2 out of 7 subjects who used this strategy found issues after the first iteration (cf. Table 4, subjects S3 and S5). For $P_2$, the subjects that got an overview of the model by either looking at the structure of the model or reading the labels of the activities already marked quality problems in this phase (cf. Table 4, subjects S4, S5, S7, and S11). In detail, 3 out of 9 subjects marked one issue, one subject identified even 7 quality problems while getting an overview. The remaining subjects got an overview of the model by reading the pool and lane descriptions of $P_2$ and therefore were not able to find any issues in this phase (cf. Table 4, subjects S2, S3, S8, S9, and S10). Like in $P_1$, subjects identified most issues in the first inspection iteration, i.e., only one subject marked one issue afterward (cf. Table 4, subject S7).

For example, Fig. 3 shows the temporal order how 2 subjects identified issues in $P_2$. After looking at the lane and pool descriptions, subject S3 identified all issues in one iteration. The dashed line representing the order in which the subject marked quality problems is almost linear and therefore shows that he read the model rather sequentially like the majority of subjects did. Small deviations in the line representing subject S3 from the perfectly linear order are due to alternative ways to sequentially read the model. The line representing subject S7 has quite a distinct shape. The line is almost linear for the first 7 issues, then a jump can be observed at issue 8. From there the line again has a nearly linear shape until the jump at position 22. Comparing this line to Table 4, subject S7 identified 7 issues while getting an overview of the model. Then the subject started to check for quality problems from the start event, leading to the spike at position 8 in the diagram. He marked 14 quality problems in the first iteration. Afterward, as the jump at position 22 indicates, he spotted one issue in a second iteration, before stopping to inspect the model.

#### Strategy $T_2$

Subjects adopting this strategy renounced getting an overview of the process model and directly started with reading the model while checking for quality issues. Regarding $P_1$ (cf. Fig. 1), subjects began to read the model: “So let’s start here. So we’re starting with muesli to select, then we order the muesli.”). For $P_1$ 3 out of 12 subjects (25 %) and for $P_2$ only 1 out of 12 subjects (8.33 %) used strategy $T_2$. Note that $P_2$ (45 activities) was much larger than $P_1$ (13 activities). The subjects that used this strategy for $P_1$ but not for $P_2$ abandoned it for strategy $T_1$, i.e., they got an overview of $P_2$ before they started identifying quality issues (cf. Table 4, subjects S2 and S4).

All 3 subjects that read $P_1$ directly without getting an overview of the model first inspected it in one run. The one subject that applied this strategy for $P_2$ (subject S1) used two iterations, but marked all issues in the first iteration. Figure 4 depicts the temporal order of this subject’s identified quality problems in $P_2$. The line is almost linear for the first 7 issues, then a drop can be observed at issue 8, followed by a jump at position 10. Afterward, the line again has a nearly linear shape. In particular, subject S1 started reading the model sequentially like the majority of subjects did. However, in relation to other subjects, he decided at a data-based exclusive gateway to follow another path than the majority of the subjects did, leading to the drop at position 7. After inspecting this part of the model, he continued with the other path of the data-based exclusive gateway, resulting in the spike at position 10. Finally, he finished reading the model in a sequential reading order. In summary, subject S1 read the whole model sequentially, but in a different way than the majority of the subjects.

#### Strategy $T_3$

2 out of 12 subjects (16.67 %) preferred to identify quality issues in $P_1$ and $P_2$ by checking off a mental list of possible quality problems, i.e., looking for specific types of quality problems across the model. For example, one subject started analyzing $P_1$ (cf. Fig. 1) by checking properties of message flows: “Well first I check if there are messages in between of the lanes because that’s not allowed.” Afterward, she got an overview of the process model by reading the pool descriptions: “Now I check what kind of pools there are...” Then, she validated if all labels were conform to the verb-object style: “Now I check for the labels [...] and there should always be a verb and a subject.” She moved on to checking the logic of the whole process: “Alright now I go over the logic...Alright, first the customer selects the muesli...” She continued to look for sequence flow line crossings and controlling the correctness of loops: “I check for crossings which are not necessary...but there aren’t. And I check for loops...” Afterward, she checked if the placement of activities were correct regarding their lanes: “The payment is in the accounting...that’s all production...product management...yes, that’s customer activities.” After searching for message flow crossings, she finished her analysis of $P_1$ by a quick scan of the whole model.

Regarding the reading order of the 2 subjects that applied $T_3$, they did this in 2 up to 9 iterations, using a new iteration for each quality problem they were looking for. Figure 5 shows the order in which both subjects identified quality problems in $P_2$. The dashed line representing the order in which subject S6 spotted quality problems starts by a small jump at position 2, followed by a small drop at position 3. Afterward, the line is almost linear. The small agitations at the beginning of the inspection of $P_2$ can be explained by subject S6 searching for incorrect labels. Then he decided to look for issues at the control flow, resulting in a line that shows him reading the model rather sequentially like the majority of subjects did. The line representing subject S12 has quite a different shape due to many jumps and drops. The mental list of subject S12 was more extensive than the mental list of subject S6, resulting in many iterations and therefore a very spiky line. However, during any iteration subject S12 inspected the model along the sequence flow (e.g., from position 16 to 19 where the labels of the activities were checked).

### $RQ_{2}$: What are the challenges in identifying quality problems in BPMN process models?

To answer this research question, we differentiated between true positives, false negatives and non-issues. Table 5 gives an overview of these issues. In particular, in $P_1$ 67 out of 168 quality problems (41.67 %) were marked, i.e., 101 issues remained unnoticed (58.33 %). In more detail, 25 out of 60 syntactic (41.67 %), 24 out of 36 semantic (66.67 %), and 18 out of 72 pragmatic issues (25.00 %) were found. In turn, 35 syntactic, 12 semantic, and 54 pragmatic quality problems remained unidentified. In addition, 42 non-issues were mentioned, i.e., 29 syntactic, 4 semantic, 1 pragmatic, and 8 additional non-issues were marked. Subjects identified more quality problems in $P_2$, i.e., 199 out of 300 issues (66.33 %) were spotted, resulting in 101 problems that were not mentioned (33.67 %). In particular, 95 out of 108 syntactic (87.96 %), 31 out of 48 semantic (64.58 %), and 73 out of 144 pragmatic issues (50.59 %) were marked, resulting in 13 syntactic, 17 semantic, and 71 pragmatic false negatives. Additionally, 42 non-issues were mentioned, i.e., 15 syntactic, 10 semantic, and 8 pragmatic non-issues were spotted.

**Table 5:** Number of true positives, false negatives and false positives (i.e., non-issues) per process model

	$P_1$	$P_2$
True positives	67 out of 168 (41.67 %)	199 out of 300 (66.33 %)
Syntactic true positives	25 out of 60 (41.67 %)	95 out of 108 (87.96 %)
Semantic true positives	24 out of 36 (66.67 %)	31 out of 48 (64.58 %)
Pragmatic true positives	18 out of 72 (25.00 %)	73 out of 144 (50.59 %)
False negatives	101 out of 168 (58.33 %)	101 out of 300 (33.67 %)
Syntactic false negatives	35 out of 60 (58.33 %)	13 out of 108 (12.04 %)
Semantic false negatives	12 out of 36 (33.33 %)	17 out of 48 (35.42 %)
Pragmatic false negatives	54 out of 72 (75.00 %)	71 out of 144 (49.41 %)
False positives	42	42
Syntactic false positives	29	15
Semantic false positives	4	10
Pragmatic false positives	1	8
Other false positives	8	0

We investigated the specific quality issues in detail^4^ and observed that subjects correctly identified some quality problems more often than others (cf. Table 6). To provide a better overview, Table 6 divides the correctly identified quality issues into two groups, issues that were frequently identified (i.e., issues that were marked by at least 6 out of 12 subjects in over 60 %) and issues that gained less attention (i.e., issues that were marked by at most 7 out of 12 subjects in <40 %).

**Table 6:** Overview of identified true positives

	Syntactic	Semantic	Pragmatic
Easily identified issues	Parallel gateways and data-based exclusive gateways At least 8 out of 12 subjects marked by 87.50 %	Activities out of context (obvious) At least 10 out of 12 subjects marked by 91.67 %	Implicit gateways At least 6 out of 12 subjects marked by 75.00 %
		Activities out of context (difficult) At least 6 out of 12 subjects marked by 62.5 %	Labels At least 6 out of 12 subjects marked by 63.33 %
Issues that gained less attention	Event-based exclusive gateways At most 3 out of 12 subjects marked by 25.00 %	Semantic issues At most 4 out of 12 subjects marked by 29.17 %	Message flows description 1 out of 12 subjects
	Message flows 2 out of 12 subjects		Line crossings At most 3 out of 12 subjects marked by 16.37 %
			Reverse sequence flow direction At most 7 out of 12 subjects marked by 38.89 %

**Table 7:** Overview of challenges in identifying quality problems

Challenge	Syntactic	Semantic	Pragmatic
Lack of BPMN knowledge			
False negatives	3 Subjects	1 Subject	–
	5 Times mentioned	Once mentioned	
False positives	29 Different issues (44 total)	–	–
Lack of domain knowledge			
False negatives	–	1 Subject	–
		Once mentioned	
False positives	–	5 Different issues (8 total)	6 Different issues (6 total)
Unclear inspection criteria			
False negatives	–	–	3 Subjects
			3 Times mentioned
False positives	–	–	7 Different issues (9 total)
Context			
False negatives	4 Different issues (8 total)		
Overlooked issues			
False negatives	–	3 Subjects	4 Subjects
		4 Times mentioned	4 Times mentioned

In detail, all syntactical errors with respect to parallel and data-based exclusive gateways (1 in $P_1$ and 9 in $P_2$) were identified by at least 8 out of 12 subjects (66.67 %). Overall, 87.50 % of these issues were marked. Also, pragmatic issues relating to gateways, i.e., implicit gateways (2 in $P_2$), were identified by at least 6 out of 12 subjects (50.00 %, overall 75.00 % issues marked).

We inserted in $P_1$ 2 and $P_2$ 3 superfluous activities. For 3 of these activities it was very obvious that they were out of context, i.e., we named them “go for a smoke,” “complain to boss” and “discuss where to go for lunch.” These activities where identified by almost all subjects (at least 10 out of 12, 75.00 %). Overall, these activities were marked to 91.67 %. Another superfluous activity labeled “call Claudia” was identified by 9 out of 12 subjects (75.00 %). For our last superfluous activity, i.e., “check stock market news” it was less obvious that it was out of context. The issue was discovered by 6 out of 12 subjects (50.00 %).

With respect to labels, $P_1$ and $P_2$ both contain 2 activities with non-intention-revealing labels, $P_2$ additionally 1 activity that is not according to verb-object style. Overall, 63.33 % of these quality issues were identified by at least 6 out of 12 subjects (50.00 %).

While the above-mentioned issues were identified by most subjects, other quality problems gained less attention. For example, all syntactical errors relating to event-based exclusive gateways (2 in $P_1$) were found by at most 3 out of 12 subjects (25.00 %). In total, only 25.00 % of these errors were marked.

With respect to message flows, in $P_1$, we deleted from 5 sending tasks the filled envelope marker that distinguished these tasks from normal activities. Only 2 out of 12 subjects (16.67 %) marked each one of those 5 activities. Moreover, 1 out of 12 subjects mentioned that not all message flows have a line description.

In addition to 5 superfluous activities, we added 1 semantic issue to each model. In $P_1$, it can happen that the customer has to pay more than once for his muesli. In $P_2$, we switched the labels of the lanes. Of these issues, 29.17 % were marked by at most 4 out of 12 subjects (33.33 %).

Regarding line crossings, we introduced 1 unnecessary line crossing to $P_1$ and 2 to $P_2$. However, only 3 out of 12 subjects (25.00 %) ever mentioned a line crossing. One of them identified all line crossings because she used a strategy to identify quality issues where she explicitly looked out for crossings of the sequence and message flows (cf. Sect. 5.1, $T_3$). Therefore, only 16.37 % of line crossings in $P_1$ and $P_2$ were mentioned.

In $P_2$, at 3 places in the model the sequence flow is from right to left instead of the other way round. These issues were identified to 38.89 % by at most 7 out of 12 subjects (58.33 %).

#### Challenges

In order to obtain a better understanding of the challenges that arose, we analyzed the spotted non-issues (i.e., false positives) as well as the feedback from the subjects about the false negatives (i.e., while looking at the models with marked quality issues subjects mentioned reasons why they were not able to find specific quality issues). With the help of grounded theory methods, we abstracted different challenges in identifying quality problems. In particular, Table 7 gives an overview of the challenges we observed.

#### Lack of BPMN knowledge

One challenge when inspecting BPMN process models relates to the lack of BPMN knowledge to determine if an issue is a serious quality problem. We found 3 subjects explaining that they were not able to identify a specific issue because of lack of BPMN knowledge. Taking a closer look, these subjects mentioned altogether 6 times that they had troubles because they have insufficient knowledge about BPMN, e.g., “Honestly I think I have some problems with understanding these modeling elements [event-based exclusive gateways], because the models I’ve created and models I’ve seen so far have mainly used activities, and/or [gateways], just simple processes without all those.”

In addition, we observed 29 different occurrences of syntactical non-issues being identified (44 in total), which were marked because of lack of BPMN knowledge. For instance, a subject claimed that the throw message event “send notification” in the “product management” lane of $P_1$ must be followed by two activities, even though there is no need for that: “Here is an error because there should be at least two tasks after such an event [throw message event].”

#### Lack of domain knowledge

Like BPMN knowledge, knowledge about the domain is also crucial for determining if a model represents reality. For instance, one subject had serious understandability issues because she did not recognize that the activity “check stock market news” in $P_2$ is out of context. She got influenced by this activity to the extent that she thought that the whole new product development process is about stocks: “Stock market news. It seems like that’s not a product, it’s more like, stocks...That would make sense, because then you wouldn’t have a development of the product itself.” Therefore, she concluded that the parts of the model about the materials for the product development are incorrect, because there is no need for materials if the process is about stocks: “Are materials available...it’s not are stocks available. That’s really bad! That’s awful.”

Moreover, semantic non-issues were identified due to a lack of domain knowledge. We found 5 different occurrences of semantic non-issues being marked (8 in total). For example, at $P_2$ two subjects did not see any benefit in ordering booth displays to advertise a new product because they did not fully understand the intention behind this activity. Therefore, they identified the activity “order booth displays” as superfluous: “‘Order booth displays’, that’s also a semantic error. There’s no meaning for me.”

In addition to semantic non-issues, 6 pragmatic non-issues were also marked by subjects because activity labels were not intention-revealing for them, i.e., subjects did not understand the meaning behind a label and marked the activity as an issue. For example, in $P_2$ one needs to create an entry for a new material supplier in a database. One subject marked the activity “create supplier in database” as an error because it does not represent her view of the domain: “This [‘create supplier in database’] should also be a compound action because it seems different level of granularity. I mean ‘create supplier in database’ seems like you should first just find one and do some discussion and stuff. You just don’t have a supplier ready.”

#### Unclear inspection criteria

One reason for not finding quality issues could be traced back to subjects not agreeing on particular pragmatic quality issues. While 3 subjects were aware about these types of quality issues in general, they felt that respective occurrences are not hampering understandability. In detail, one subject agrees in general that line crossings might be an issue, but could not see how to prevent a line crossing in $P_1$ and claimed that there is no issue with this particular occurrence of a line crossing: “That one for me is no problem at all because you cannot avoid it.” Another subject mentioned that a line crossing issue in $P_2$ does not bother him: “That’s for me...it’s ok like this, doesn’t make it harder to read.” A third subject argued that reverse sequence flow directions in $P_2$ are not issues, but rather a personal matter: “Then we have the flow going from right to left. [...] That’s something personal I think. I mean I wouldn’t start modeling a model from right to the left, I also wouldn’t start a model from the top to the bottom, but actually a number of modelers is modeling from the top to the bottom and I think this is a personal thing. I actually didn’t even realize that it’s going from the right to the left. I think this is also a little bit of a space issue. I mean in this example it would have been enough space to model from the left to the right so yes, it’s actually probably nicer if you can always read in one direction, but in that area it didn’t disappoint me, so for me it was ok.”

Additionally, we identified 7 different occurrences of pragmatic non-issues being marked (9 in total) which were caused by lack of knowledge about pragmatic criteria. For example, subjects criticized labels even though they were conform to verb–object style, e.g., in $P_2$ the activity “perform user acceptance testing” was marked as an issue, even though it is correct: “‘Perform user acceptance testing’ is a pragmatic error.”

#### Context

**Fig. 6:**
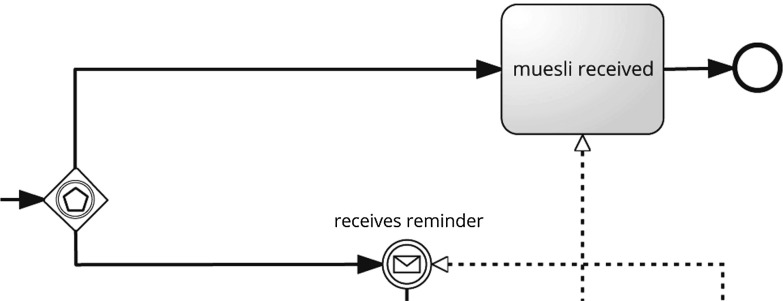
Snippet of $$P_1$$

Another challenge was that subjects identified non-issues without considering the context of the quality problem. In particular, subjects marked 4 different non-issues (8 in total) resolving the problem locally, but the issue was still incorrect globally. For example, at $P_1$ there is an event-based exclusive gateway which is followed by one event and one activity (cf. Fig. 6), which is incorrect because an event-based exclusive gateway must be followed only by events. As the activity should be an event, it is also named like one, namely “muesli received.” Locally, the label is not conform to verb–object style, which was mentioned by 3 subjects (e.g., “Here it’s ‘muesli received’. It should more be...it’s in the past, so it should be ‘receive muesli’.”). However, these subjects failed to identify the global issue, i.e., they did not see that the activity should be an event. We consider these kinds of issues as non-issues, because even though the subjects resolved the local quality problem, the global issue still remained.

**Table 8:** Number of correctly classified issues

	Syntactic issues	Semantic issues	Pragmatic issues
$P_1$	12 out of 20 (60 %)	13 out of 21 (61.90 %)	12 out of 12 (100 %)
$P_2$	63 out of 79 (79.75 %)	15 out of 22 (68.18 %)	42 out of 57 (73.68 %)

#### Overlooking quality problems

One reason why issues gained rather less attention was because the subjects simply overlooked these issues. In particular, 6 subjects stated this reason at one or two not identified issues while inspecting the models with marked quality problems. In more detail, 4 times it was directly mentioned that the issue was overlooked, e.g., “And then what about this one [issue], ‘check stock market news’. I haven’t read that part actually.” Subjects stated 3 times that they were focused too much on another issue, e.g., “Actually, I was thinking about line crossing, but I didn’t talk about it and I also didn’t mark it because I was thinking about this stuff down there [pointing at another issue].” Another subject mentioned that she was not able to see the reverse sequence flow direction because the model was printed on paper: “Also, the reverse flow. I just find it really difficult to spot if you have it on paper. It’s very difficult to say it shouldn’t be here. [...] Maybe it would be easier if you could...if it were not on paper.”

### $RQ_{3}$: Can classifying issues with the quality dimensions of the SEQUAL framework help humans when inspecting a BPMN process model?

#### Lack of clarity about classification

As described in Sect. 4, we inspected if quality problems were classified correctly. Out of 221 classified issues, 157 were correctly classified (74,41 %). For more details, see Table 8.

In general, there was a high percentage of correctly classified issues. However, in some cases subjects had difficulties. In particular, the differentiation between semantic and pragmatic quality issues caused problems for some subjects, e.g., “I really have problems to separate pragmatic and semantic. Pragmatic is if the naming of the certain activity is wrong and semantic is...[continuing on a different matter].” We could also observe these difficulties in the transcripts for activities that are superfluous or out of context, i.e., they were related to either the semantic or the pragmatic quality dimension. For example, some subjects correctly identified the activity “check stock market news” in $P_2$ as a semantic issue because it is out of context: “‘Check stock market news’...So I don’t know why we check the stock market news...After we create the purchase requisition...This here [strikes out ‘check stock market news’] makes no sense, we can skip this one. And this would be again something out of the context, it’s a semantic error, process out of context.” Other subjects labeled it incorrectly as a pragmatic issue for the same reason: “‘Check stock market news’, ok, that would also be pragmatic. Because, I mean maybe I got it wrong but stock market would not be a stock you would refer to, no, I think that it’s out of context here.”

In general, issues about labels or the layout were correctly referred to as pragmatic issues, which some subjects even called “layout issues.” For example, in $P_2$ we used the activity “develop product” as implicit gateway instead of a data-based exclusive gateway. One subject classified this issue as a “layout or pragmatic” issue: “Here [pointing at an implicit gateway] once again we should have another XOR. Otherwise, we have two incoming branches in one activity. This would be a layout or pragmatic error.”

#### Use of classification

We identified 3 manners in which classification was addressed, i.e., reactive classification, proactive classification without reasoning and proactive classification with reasoning.

Reactive classification. The subject identified an issue and was asked to classify it by the supervisor, so he/she did it. For instance, one subject needed to be reminded to classify the non-intention-revealing label “reminder” in $P_1$: “‘Reminder’ is also not a really good name for an activity, so it should be either ‘send reminder’ or something like that. [Supervisor: Which kind of error?] Ok, this would be...I guess a pragmatic error I would say.”

*Proactive classification without reasoning* The subject used the classification proactively, but did not provide any explanation why a certain quality dimension was chosen. For example, a subject marked and classified a deadlock in the production lane in $P_1$ by just stating that the first gateway is wrong and without giving further explanation why it would be a quality problem or a syntactical issue: “So this [pointing at a data-based exclusive gateway] has to be an AND gateway I think, which is a syntactical one [error].”

*Proactive classification with reasoning* The subject identified an issue and reasoned about it through the classification. Using the example from before, even though one subject incorrectly classified the deadlock as a syntactic and pragmatic quality problem, he correctly explained that the data-based exclusive gateway should be a parallel gateway because otherwise the customer either gets muesli without an invoice or gets an invoice for muesli he did not receive: “It doesn’t makes sense here [circles data-based exclusive gateway]. That’s a syntactical and pragmatic error because you cannot just produce the meal or send invoice. When you’ve produced everything he will get an invoice, so that’s a mistake here.”

In general, subjects rather proactively classified quality issues than having to be asked about them by the supervisor. From 211 classified issues, 41 times (19.43 %) quality problems were classified by reactive classification. Subjects applied the second manner, i.e., proactive classification without reasoning, 91 times (43.13 %). Subjects used the third manner, i.e., proactive classification with reasoning, 79 times (37.44 %). For $P_1$ the dominant manner was the third one (28 out of 54 classifications), while for $P_2$ subjects favored the second manner (76 out of 158).

Apart from these manners, one subject sometimes used the issue type to classify issues. For instance, he did not state that a specific gateway is wrong, but mentioned that there is, e.g., a deadlock, and therefore the issue is syntactic: “Again, we have here a deadlock [pointing to a deadlock], which is syntactic.”

## Discussion

### Strategies

We could identify three different strategies that our subjects adopted for inspecting and identifying quality issues in BPMN process models (cf. Sect. 5.1). However, it is not clear if there are any other strategies how humans spot quality issues, so further empirical research is needed. Our findings indicate that humans that adopt either strategy $T_1$ (first getting an overview of the process model before checking it for quality issues) or strategy $T_3$ (having a mental list of possible quality problems to inspect a process model) use these strategies irrespective of the model’s size or complexity. Otherwise, it seems that humans that renounced looking over the process model before starting to read and check it (strategy $T_2$) mostly preferred to switch to strategy $T_1$. In addition, we observed that the order in which quality issues were spotted was primarily driven by the respective strategy the subjects applied for inspecting the process model and then followed the temporal order of the process.

We do not know if humans with less BPMN knowledge prefer other strategies than modeling experts. In our study, subjects adopted strategies irrespective of their demographic background, i.e., no strategy was only used by students, academics or professionals. In general, we observed several times behavior that is characteristic for experts, i.e., the presence of a systematic navigation or the usage of a mental list [39, 49].

Further, a main question is whether certain strategies are more effective. For this, our sample is too small. In addition, the question remains unsettled which strategy should be used to spot a specific kind of quality issue, or, on the contrary, which strategy should not be used to identify a specific kind of quality issue. The classification with which we manipulated the inspection process did not seem to affect the strategies taken (i.e., it did not serve as a mental list). Additionally, how strategies change when more support is provided, e.g., through checklists, remains an open question. Yet, we indicated the existence of these strategies and further research can relate to their effectiveness.

### Challenges

For each quality dimension, we observed quality issues that were spotted by a large number of subjects, but also quality problems that gained less attention (cf. Sect. 5.2). One way to deal with quality problems that gain less attention could be checklists, as they are known to be an effective method for software testing [38] or user interface evaluation (e.g., [22]). Detailed collections of possible syntactic errors are given in [24, 56]. Pragmatic issues are gathered in [58] and [46]. Semantic quality problems refer to the validity and the completeness of a model. Therefore, a checklist focusing on semantic issues needs to be domain specific.

All in all, we could identify different challenges why our subjects missed quality problems or marked non-issues (cf. Sect. 5.2). Our data also showed that the types of challenges subjects faced were closely related to the dimensions of the SEQUAL framework. One challenge was clearly lack of BPMN knowledge. This challenge mostly relates to syntactic quality issues, but never to pragmatic problems (cf. Table 7). In particular, lack of BPMN knowledge was the main reason for identified syntactic non-issues. This finding emphasizes the need of profound knowledge of the modeling language.

Further, lack of domain knowledge challenged our subjects. Lack of domain knowledge relates mostly to semantic issues (cf. Table 7). According to [34], a problem-solving task like inspecting a process model starts by a human formulating a mental image of the problem, i.e., a mental model of the domain of the process model to inspect. This mental model is then used to argue about the solution (i.e., reason why an issue is a quality problem). [39] mentions that the act of deriving a mental model of a system’s structure in a particular domain is important to a professional performance. Our findings indicate that obvious superfluous activities are identified easily. However, it seems that one unapparent superfluous activity is enough to hamper the understandability of a whole model, i.e., one subject’s mental model of $P_2$ was biased by a single superfluous activity, leading to serious understandability issues. Moreover, as described in [16], humans are not “empty vessels to be filled with model information,” but beings that integrate the processed information from the process model under inspection with their prior experience. Some subjects’ mental models were biased by their domain experience, resulting in marked non-issues (cf. example of creating a new supplier in a database in Sect. 5.2). Therefore, an additional textual process description or an approach like literate modeling [1] which allows to explain certain design decisions might mitigate the risk of a biased mental model.

Some subjects were challenged by their lack of clear inspection criteria regarding pragmatic issues (cf. Table 7). For instance, one subject argued that the direction of a sequence flow in a model is a personal matter. [39] agrees with this opinion and states that personal style and individual skill are influencing the comprehension of a graphical representation. This research is currently picked up by [6], which investigates the visual layout of process models. In particular, it focuses on layout properties that are meaningful to humans and suggests a set of measures for operationalizing these layout properties, which are a key for clearer inspection criteria.

Considering the context of a possible quality problem posed a challenge. The importance of context is shown, for example, in [7], which states that relevant contextual knowledge is a requirement for understanding prose passages. One way to support subjects in overcoming troubles with the context of quality issues could be a test-driven approach [3], i.e., a manual step-by-step validation of the process model. While there exists an implementation for declarative process models [62], this is not the case for BPMN process models. Also, current proposals for validation of BPMN models (e.g., [26]) focus on syntactic issues only.

Another problem was that subjects overlooked quality problems. Research on perception indicates that in visual processing the ability to focus on the most relevant information in a graphic representation is crucial to a expert performance [39]. However, in a model inspection process all parts of a process model should be considered, not only the most relevant ones. A way to deal with this problem could be a systematic approach, e.g., either the use of a test-driven approach, or using a checklist for quality problems.

### Classification

Our study results indicate 3 manners in which classification was addressed (cf. Sect. 5.3). In the case of reactive classification (i.e., the supervisor asked the subject to classify a quality problem), the classification did not contribute positively to identifying quality issues in process models, as otherwise the subject would not have classified the issues at all. In case of proactive classification without reasoning (i.e., the subject classified a quality issue without further explanation), it is not clear whether or not the classification helped in identifying a specific issue. Proactive classification with reasoning (i.e., the subject marked a quality issue and classified it while reasoning about the classification) indicates a positive contribution to the identification of quality problems. According to the cognitive load theory [8], the use of schemas that allow the classification of multiple elements as a single element can reduce the burden on the limited capacity of working memory [36]. In particular, [55] argues that classification can ease understanding and conceptualizing process behavior. Also, [37] states that classification helps inferring by serving as a cognitive schema to which current information is mapped. In summary, classification can help to reduce cognitive load and build a mental model of the process model domain (i.e., ease the comprehension and abstraction of the process model). For the majority of cases in our study, it is not clear if there is a positive contribution of the classification (i.e., 91 of 211 classified issues were classified by proactive classification without reasoning). However, in 79 of 211 cases the classification had a positive influence on identifying a quality problem (i.e., the issues were classified with proactive classification with reasoning).

Even though the majority of issues was correctly classified, some subjects had difficulties to distinguish between semantic and pragmatic quality issues. [57] gives a good overview of heuristics and biases humans use when judging under uncertainty. As classification can have a positive influence on cognitive load and model understandability, we want to emphasize the importance of being able to classify quality issues to teachers and educators of future system analysts.

### Limitations

This study has to be viewed in the light of several generalization limitations. Note that generalization was not aimed at. Rather, the study is exploratory, highlighting future research directions. Quantitative and more focused studies are still to follow.

The detection of quality issues presumably also depends on the support that is offered. In the sense of notational support, models may be specifically designed for a group of stakeholders, making the models particularly suitable for understanding and thus detecting quality issues. In this work, we focused on BPMN, i.e., a notation that is typically taught to a large group of stakeholders [42]. Even so, our findings might be difficult to generalize to more specifically tailored notations [43]. In the sense of computer support, such as scrolling or syntax highlighting, we specifically decided to conduct our study without tool support to establish a baseline further studies can be compared against. In addition, we aim at a better understanding of the way users interact with the process model and to use the obtained insights to inform the design of better tool support that takes the user’s behavior into account. Caution should be taken when transferring our insights to tool-supported process models, but basic applicability is mostly expected. We would like to stress that these decisions were made deliberately and this study should be seen as exploratory, highlighting future research directions.

Regarding further limitations, the study is limited to two process models created with BPMN (cf. Sect. 3), but BPMN is a de facto standard [42], the models are realistic in size [15] and designed to cover certain modeling elements [33]. In addition, both process models contain relevant quality issues of all three dimensions (cf. Sect. 2).

Another limitation is relating to domain knowledge. As shown in Table 3, it seems that knowledge about the domain varied. We did not provide textual domain descriptions about the processes since this might have strongly affected the inspection process and obscure the strategies by a linear comparison between the text and the model.

Furthers, it should be noted that the number of subjects in the study is relatively low (12 subjects). Nevertheless, it is noteworthy that the sample size is not unusual for this kind of empirical investigation due to the substantial effort to be invested per subject [10, 35]. Also, half of the participating subjects were students. However, all subjects indicated profound background in business process management (cf. Table 3).

## Related work

The goal of this study is to investigate how humans identify quality issues in BPMN process models. Errors at the syntactic level can be automatically detected for a large class of process models using verification techniques, which also support the incremental validation of process models [26]. The identification of semantic errors can only be partially automated [54]. For instance, [5] describes a two-step procedure for measuring process model quality at the semantic level by comparing a process model with a reference model. First, activities present in the process model must be mapped to the activities of the reference process model, e.g., using measures like the Levenshtein distance for activity labels [27] or combining edit distances measures with the detection of synonyms [11]. Similarly, the ICoP framework [59] provides means to automatically detect potential activity matches between process models. After establishing an activity mapping, the similarity to the reference model can be assessed [5] by measuring edit distances between graphs, e.g., [11], focusing on causal dependencies of activities, e.g., [60]. In turn, [12] suggests a technique for detecting redundant process fragments and for automatically extracting them to subprocesses. Moreover, techniques for modularizing large process models and for automatically labeling the extracted subprocess fragments are proposed in [51]. Further, [40] looks for the layout aesthetic that has the greatest effect on understandability, while [6] search for layout properties that are meaningful to humans. [52] offer an overview of selected BPMN tools regarding their support for modeling guidelines.

Similar to the study described in this paper, [21] describe a study on how IT professionals inspect two types of diagrams (entity-relationship diagrams and data flow diagrams), focusing on cognitive theory. The study’s results indicate that the way how humans process information impacts processing success. Another study described in [31] specifies understandability as a representative for quality of process models and investigates factors that might influence the comprehension of process models. In contrast to our study, process models modeled in an EPC-like notation without events were chosen for the study. Another difference is that the attention is on personal factors and model characteristics, instead of the modeler’s behavior, i.e., the way how humans inspect process models. While the study described in this paper centers on how quality issues are identified by humans, [19] report from a case study in cooperation with a large Norwegian oil company that focuses on how syntactic quality influences pragmatic quality in BPMN enterprise process models.

This paper takes a first step toward an in-depth understanding of how BPMN process models are inspected, what strategies are taken, and what challenges are involved.

## Summary and outlook

While some quality issues can be detected automatically, many others cannot. Even though human inspection of process models is still essential [53], this manual inspection is currently not supported. This paper takes a first step toward an in-depth understanding of how BPMN process models are inspected, what strategies are taken, and what cognitive processes are involved. The presented exploratory study investigates the strategies taken by humans when inspecting BPMN process models, the kinds of challenges they face while identifying quality problems, and how marked quality issues were classified. Our qualitative analysis shows that humans adapt different strategies on how to identify quality issues. We observed for each quality dimension quality issues that were spotted by a large number of subjects, but also quality issues that gained less attention. We point out different challenges why some quality problems were not spotted and why issues that were no quality problems were marked. Moreover, we identified different manners how quality issues were classified. Further, we also indicate other research questions that can be investigated using this approach. In this way, this paper constitutes another building block toward a more comprehensive understanding of how humans inspect process models, guiding model inspection support for humans, as well as pointing out typical challenges to teachers and educators of future system analysts.

Future research can build upon these initial findings by performing more comprehensive studies. In particular, future studies should contain question about the participants’ knowledge on any quality framework or classifications. In addition, which quality issues should be supported with appropriate tool support and which parts of a modeling notation are challenging while creating or maintaining a process model remain open questions. Likewise, we plan to extend our research focus by additionally asking practitioners and business managers to inspect process models for quality issues.

## Footnotes

1 The study’s material can be downloaded from: http://bpm.q-e.at/QualityIssuesBPMN.

2 A high resolution of this figure can be downloaded from: http://bpm.q-e.at/QualityIssuesBPMN.

3 A high resolution of both models can be downloaded from: http://bpm.q-e.at/QualityIssuesBPMN.

4 The study’s data can be downloaded from: http://bpm.q-e.at/QualityIssuesBPMN.

## References

[1] Arlow, J., Emmerich, W., Quinn, J.: Literate modelling—capturing business knowledge with the UML. In: Proceedings UML’98, pp. 189–199 (1999)

[2] Bassey M (1999). Case Study Research in Educational Settings. Doing Qualitative Research in Educational Settings.

[3] Beck K (2002). Test Driven Development: By Example.

[4] Becker, J., Rosemann, M., Uthmann, C. V.: Guidelines of business process modeling. In: Business Process Management, Models, Techniques and Empirical Studies, pp. 30–49 (2000)

[5] Becker M, Laue R (2012). A comparative survey of business process similarity measures. Comput. Ind..

[6] Bernstein, V., Soffer, P.: How does it look? Exploring meaningful layout features of process models. In: Proceedings of Advanced Information Systems Engineering Workshops—CAiSE 2015 International Workshops, Stockholm, 8–9 June 8-9 2015, pp. 81–86 (2015)

[7] Bransford J, Johnson M (1972). Contextual prerequisites for understanding: some investigations of comprehension and recall. J. Verbal Learn. Verbal Behav..

[8] Chandler P, Sweller J, Associates LE (1991). Cognitive load theory and the format of instruction. Cognition and Instruction.

[9] Corbin J, Strauss A (2007). Basics of Qualitative Research: Techniques and Procedures for Developing Grounded Theory.

[10] Costain, G. F.: Cognitive Support During Object-oriented Software Development: The Case of UML Diagrams. PhD thesis, University of Auckland (2007)

[11] Dijkman RM, Dumas M, van Dongen BF, Käärik R, Mendling J (2011). Similarity of business process models: metrics and evaluation. Inf. Syst..

[12] Dumas M, García-Bañuelos L, La Rosa RUM (2013). Fast detection of exact clones in repositories of business process models. Inf. Syst..

[13] Dumas M, Rosa M, Mendling J, Reijers H (2013). Fundamentals of Business Process Management.

[14] Ericsson KA, Simon HA (1993). Protocol Analysis: Verbal Reports as Data.

[15] Fahland D, Favre C, Jobstmann B, Koehler J, Lohmann N, Völzer H, Wolf K, Dayal U, Eder J, Koehler J, Reijers H (2009). Instantaneous soundness checking of industrial business process models. Proceedings of Business Process Management, 7th International Conference, BPM 2009, Ulm, Germany, 8–10 Sept 2009.

[16] Gemino A, Wand Y (2003). Evaluating modeling techniques based on models of learning. Commun. ACM.

[17] Gschwind, T., Pinggera, J., Zugal, S., Reijers, H., Weber, B.: A Linear Time Layout Algorithm for Business Process Models. Technical Report RZ3830, IBM Research (2012)

[18] Haisjackl, C., Pinggera, J., Soffer, P., Zugal, S., Lim, S., Weber, B.: Identifying quality issues in BPMN models: an exploratory study. In: Proceedings of BPMDS’15, pp. 217–230 (2015)

[19] Heggset, M., Krogstie, J., Wesenberg, H.: The influence of syntactic quality of enterprise process models on model comprehension. In: Proceedings of the CAiSE 2015 Forum at the 27th International Conference on Advanced Information Systems Engineering co-located with 27th International Conference on Advanced Information Systems Engineering (CAiSE 2015), Stockholm, 10th June, pp. 89–96 (2015)

[20] Leopold JMH, Smirnov S (2012). On the refactoring of activity labels in business process models. Inf. Syst..

[21] Hungerford BC, Hevner AR, Collins RW (2004). Reviewing software diagrams: a cognitive study. IEEE Trans. Softw. Eng..

[22] Johnson G, Clegg C, Ravden S (1989). Towards a practical method of user interface evaluation. Appl. Ergon..

[23] Khatri V, Vessey I, Ramesh PCV, Park S-J (2006). Understanding conceptual schemas: exploring the role of application and IS domain knowledge. Inf. Syst. Res..

[24] Koehler, J., Vanhatalo, J.: Process anti-patterns: how to avoid the common traps of business process modeling. Technical report, IBM ZRL Research Report 3678 (2007)

[25] Krogstie J (2012). Model-Based Development and Evolution of Information Systems: A Quality Approach.

[26] Kühne S, Kern H, Gruhn V, Laue R (2010). Business process modeling with continuous validation. JSEP.

[27] Levenshtein W (1966). Binary codes capable of correcting deletions, insertions and reversals. Sov. Phys. Dokl..

[28] Mendling J (2008). Metrics for Process Models: Empirical Foundations of Verification, Error Prediction and Guidelines for Correctness.

[29] Mendling, J.: Empirical studies in process model verification. In: Transactions on Petri Nets and Other Models of Concurrency II, pp. 208–224. Springer (2009)

[30] Mendling J, Reijers H, Recker J (2010). Activity labeling in process modeling: empirical insights and recommendations. Inf. Syst..

[31] Mendling, J., Reijers, H. A., Cardoso, J.: What makes process models understandable? In: Proceedings of BPM’07, pp. 48–63 (2007)

[32] Mendling J, Reijers HA, van der Aalst WMP (2010). Seven process modeling guidelines (7pmg). Inf. Softw. Technol..

[33] Muehlen, M. Z., Recker, J.: How much language is enough? Theoretical and practical use of the business process modeling notation. In: Proceedings of the 20th International Conference on Advanced Information Systems Engineering, CAiSE ’08, pp. 465–479. Springer-Verlag, Berlin (2008)

[34] Newell A (1972). Human Problem Solving.

[35] Nielsen J (1994). Estimating the number of subjects needed for a thinking aloud test. Int. J. Hum. Comput. Stud..

[36] Paas F, Tuovinen JE, Tabbers H, Van Gerven PWM (2003). Cognitive load measurement as a means to advance cognitive load theory. Educ. Psychol..

[37] Parsons J, Wand Y (2008). Using cognitive principles to guide classification in information systems modeling. MIS Q..

[38] Perry W (2006). Effective Methods for Software Testing.

[39] Petre M (1995). Why looking isn’t always seeing: readership skills and graphical programming. Commun. ACM.

[40] Purchase, H.: Which aesthetic has the greatest effect on human understanding? In: Proceedings of GD’97, pp. 248–261 (1997)

[41] Recker J (2007). A socio-pragmatic constructionist framework for understanding quality in process modelling. Aust. J. Inf. Syst..

[42] Recker J (2010). Opportunities and constraints: the current struggle with bpmn. Bus. Process Manag. J..

[43] Recker, J. C., Dreiling, A.: Does it matter which process modelling language we teach or use? An experimental study on understanding process modelling languages without formal education. In: Proceedings of ACIS’07, pp. 356–366 (2007)

[44] Reijers HA, Mendling J (2011). A study into the factors that influence the understandability of business process models. IEEE Trans. Syst. Man Cybern. Part A.

[45] Rittgen, P.: Quality and perceived usefulness of process models. In: Proceedings of SAC’10, pp. 65–72 (2010)

[46] Rosa ML, ter Hofstede A, Wohed P, Reijers H, Mendling J, van der Aalst WP (2011). Managing process model complexity via concrete syntax modifications. IEEE Trans. Ind. Inf..

[47] Roy S, Sajeev A, Bihary S, Ranjan A (2013). An empirical study of error patterns in industrial business process models. IEEE Trans. Serv. Comput..

[48] Scheer AW (2000). ARIS–Business Process Modeling.

[49] Schoenfeld AH, Herrmann D (1982). Problem perception and knowledge structure in expert and novice mathematical problem solvers. J. Exp. Psychol.: Learn. Memory Cogn..

[50] Schrepfer, M., Wolf, J., Mendling, J., Reijers, H.: The impact of secondary notation on process model understanding. In: Proceedings of PoEM’09, pp. 161–175 (2009)

[51] Smirnov S, Reijers H, Weske M (2012). From fine-grained to abstract process models: a semantic approach. Inf. Syst..

[52] Snoeck, M., de Oca, I. M. M., Haegemans, T., Scheldeman, B., Hoste, T.: Testing a selection of bpmn tools for their support of modelling guidelines. In: PoEM, volume 235 of Lecture Notes in Business Information Processing, pp. 111–125. Springer (2015)

[53] Soffer P, Kaner M (2011). Complementing business process verification by validity analysis: a theoretical and empirical evaluation. J. Database Manag..

[54] Soffer, P., Kaner, M., Wand, Y.: Towards understanding the process of process modeling: theoretical and empirical considerations. In: Proceedings of ER-BPM’11, pp. 357–369 (2011)

[55] Soffer P, Wand Y, Kaner M (2015). Conceptualizing routing decisions in business processes: theoretical analysis and empirical testing. J. AIS.

[56] Trcka, N., van der Aalst, W. M. P., Sidorova, N.: Data-flow anti-patterns: discovering data-flow errors in workflows. In: Proceedings of CAISE’09, pp. 425–439 (2009)

[57] Tversky A, Kahneman D (1974). Judgment under uncertainty: heuristics and biases. Science.

[58] Weber B, Reichert M, Mendling J, Reijers HA (2011). Refactoring large process model repositories. Comput. Ind..

[59] Weidlich, M., Dijkman, R., Mendling, J.: The ICoP framework: identification of correspondences between process models. In: Proceedings of CAiSE’10, pp. 483–498 (2010)

[60] Weidlich M, Mendling J, Weske M (2011). Efficient consistency measurement based on behavioral profiles of process models. IEEE Trans. Softw. Eng..

[61] Wynn MT, Verbeek HMW, van der Aalst WMP, ter Hofstede AHM, Edmond D (2009). Business process verification—finally a reality!. Bus. Proc. Manag. J..

[62] Zugal S, Haisjackl C, Pinggera J, Weber B (2013). Empirical evaluation of test driven modeling. Int. J. Inf. Syst. Model. Des..

[63] Zugal, S., Pinggera, J., Mendling, J., Reijers, H., Weber, B.: Assessing the Impact of Hierarchy on model understandability—a cognitive perspective. In: Proceedings of EESSMod’11, pp. 123–133 (2011)

[64] Zugal, S., Soffer, P., Haisjackl, C., Pinggera, J., Reichert, M., Weber, B.: Investigating expressiveness and understandability of hierarchy in declarative business process models. Softw. Syst. Model., 1–23 (2013)

